# Advanced imaging of relapse in giant cell arteritis: The role of vascular adhesion protein‐1 and [^68^Ga]Ga‐DOTA‐Siglec‐9 positron emission tomography–computed tomography

**DOI:** 10.1111/joim.20111

**Published:** 2025-06-26

**Authors:** Simon M. Petzinna, Jim Küppers, Benedikt Schemmer, Anna L. Kernder, Claus‐Jürgen Bauer, Niklas T. Baerlecken, Denada Bruci, Pantelis Karakostas, Raúl N. Jamin, Maike S. Adamson, Anja Winklbauer, Rayk Behrendt, Markus Essler, Valentin S. Schäfer

**Affiliations:** ^1^ Medical Clinic III for Oncology, Hematology, Immune‐Oncology and Rheumatology University Hospital of Bonn Bonn Germany; ^2^ Department of Nuclear Medicine University Hospital of Bonn Bonn Germany; ^3^ Department of Rheumatology & Hiller‐Research Unit Rheumatology University Hospital of Düsseldorf Düsseldorf Germany; ^4^ Department of Cardiology University Hospital of Bonn Bonn Germany; ^5^ Institute for Clinical Chemistry and Clinical Pharmacology University Hospital of Bonn Bonn Germany

**Keywords:** biomarker, giant cell arteritis, imaging, inflammation, large vessel vasculitis, vasculitis

**Abbreviations**
CRP: C‐reactive protein
CT: computed tomography
ELISA: enzyme‐linked immunosorbent assay
FDG: fluorodeoxyglucose
GCA: giant cell arteritis
IFNγ: interferon gamma
IL1β: interleukin‐1 beta
IMT: intima media thickness
MMP: matrix metalloproteinase
NK cells: natural killer cells
PET/CT: positron emission tomography–computed tomography
SUVmax: maximum standardized uptake value
sVAP‐1: soluble vascular adhesion protein‐1
TNFα: tumor necrosis factor alpha
VAP‐1: vascular adhesion protein‐1

Dear Editor,

Giant cell arteritis (GCA) is an immune‐mediated vasculitis primarily affecting medium‐ and large‐sized vessels. Although positron emission tomography–computed tomography (PET/CT) with [^18^F]fluorodeoxyglucose ([^18^F]FDG) has proven useful for assessing disease activity, persistent tracer uptake due to vascular remodeling is found in up to 80% of patients in clinical remission [1]. ^68^Ga‐labeled sialic acid‐binding immunoglobulin‐like lectin‐9 (Siglec‐9) offers potentially higher specificity for active inflammation, as Siglec‐9 functions as a ligand for vascular adhesion protein‐1 (VAP‐1) [2]. In the vasculature, VAP‐1 is expressed on vascular smooth muscle and endothelial cells, existing in both a membrane‐bound and soluble form (sVAP‐1), which is cleaved by matrix metalloproteinases (MMPs) [3]. Proinflammatory cytokines (tumor necrosis factor alpha, interferon gamma, interleukin‐1 beta) drive VAP‐1 translocation to the cell surface, where it mediates leukocyte adhesion, migration, and inflammation [1]. Recent findings suggest that [^68^Ga]Ga‐DOTA‐Siglec‐9‐PET/CT can detect vascular inflammation during GCA relapse [3, 4]. This study is the first to assess the diagnostic value of [^68^Ga]Ga‐DOTA‐Siglec‐9 PET/CT in multiple patients with relapsing GCA and to explore the roles of Siglec‐9 and VAP‐1 in GCA pathogenesis (Fig. S1).

Patients with relapsing GCA, as confirmed by a board‐certified rheumatologist, who previously fulfilled the classification criteria for GCA [5], and age‐/sex‐matched healthy controls were prospectively enrolled. The patients with active GCA underwent [⁶⁸Ga]Ga‐DOTA‐Siglec‐9‐PET/CT following intravenous injection of 135.1 ± 31.7 MBq of tracer. Low‐dose CT for attenuation correction and a whole‐body PET scan were acquired 56.2 ± 8.3 min postinjection (Supporting Information Protocol). Maximum standardized uptake values (SUVmax) were obtained for the aorta and axillary, subclavian, brachial, thoracic, and abdominal arteries. Vascular ultrasound was conducted on the superficial temporal arteries and their branches, as well as the facial, axillary, carotid, and vertebral arteries as described before [6]. Moreover, the OMERACT Giant Cell Arteritis Ultrasonography score was calculated. Levels of sVAP‐1, MMP‐2, MMP‐3, and MMP‐9 were determined by enzyme‐linked immunosorbent assay, and Siglec‐9 expression on selected peripheral blood mononuclear cells was analyzed by flow cytometry.

Eight patients with relapsing GCA and eight healthy controls were included. The corresponding demographic, clinical, laboratory, and imaging data are provided in Table S1. Tracer administration was well tolerated by all GCA patients. The [^68^Ga]Ga‐DOTA‐Siglec‐9‐PET/CT scan revealed localized, patient‐specific increases in SUVmax, most prominently in the thoracic and abdominal aorta (Fig. 1, Table S2). Vascular ultrasound showed increased intima media thickness (IMT) exceeding predefined cut‐off values in multiple vessels [6], most frequent in the axillary arteries [mean 1.28 mm (right), 1.13 mm (left)], compared to 1.05 mm (*p* = 0.20) and 0.96 mm (*p* = 0.478) prior to relapse. A significant association was found between mean SUVmax and IMT in the left axillary artery (*r* = 0.78, *p* = 0.040). Levels of C‐reactive protein (CRP) (*p* = 0.019) and MMP‐9 (*p* = 0.011) were significantly higher in GCA patients (Table S3, Fig. S2). Although sVAP‐1 did not differ significantly (*p* = 0.341), it correlated positively with CRP (*r* = 0.517, *p* = 0.040). Flow cytometry revealed significantly higher Siglec‐9 expression on intermediate monocytes (*p* = 0.002), plasma cells, plasmablasts, and naïve B cells (all *p* < 0.001), and natural killer cells (*p* = 0.032) compared to healthy controls (Table S4, Figs. S2 and S3).

**Fig. 1:**
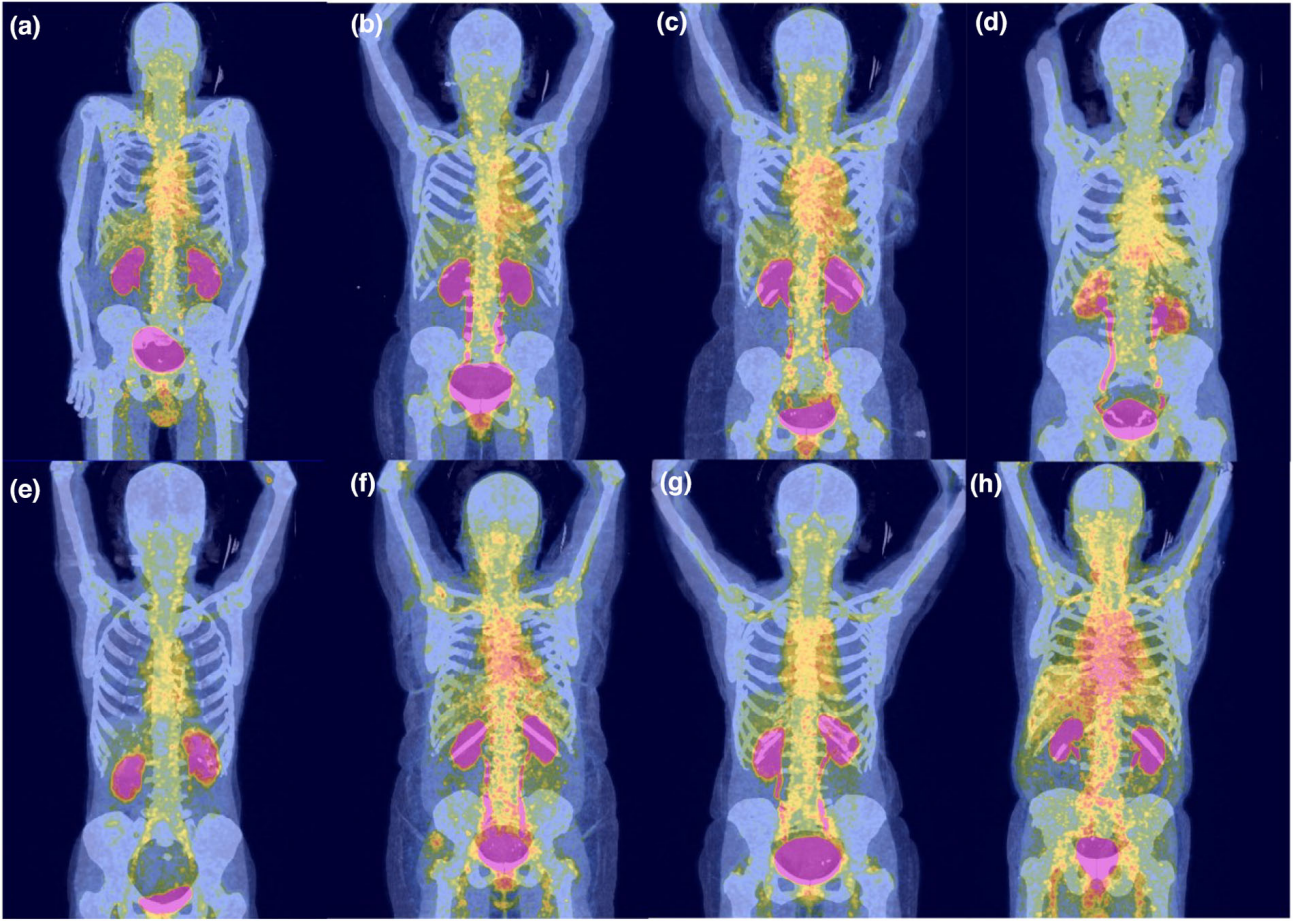
[^68^Ga]Ga‐DOTA‐Siglec‐9‐positron emission tomography–computed tomography (PET/CT) imaging findings in giant cell arteritis patients with relapse. This figure illustrates [^68^Ga]Ga‐DOTA‐Siglec‐9‐PET/CT imaging results from eight patients experiencing a relapse of giant cell arteritis. Patient‐specific tracer uptakes are revealed in various vascular regions. The PET signal intensity is represented using a standardized color scale, where brighter colors (violet > red > yellow > green) indicate higher [^68^Ga]Ga‐DOTA‐Siglec‐9 uptake, reflecting increased vascular tracer accumulation. The labels (a–h) correspond to the patient numbers listed in Tables S1–S3.

This pioneering study further supports the safety and efficacy of [^68^Ga]Ga‐DOTA‐Siglec‐9‐PET/CT [2], enabling in vivo visualization of VAP‐1 expression. The observed localized increases in SUVmax in various anatomical regions, correlating with IMT changes in vascular ultrasound, suggest local upregulation of VAP‐1 during GCA relapses. Consequently, [^68^Ga]Ga‐DOTA‐Siglec‐9‐PET/CT may facilitate the detection of acute vascular inflammation in relapsing GCA, as it addresses limitations of current PET/CT approaches by distinguishing between active inflammation and vascular remodeling.

Although our data imply a pathogenic role for endothelially expressed VAP‐1 in GCA, its exact contribution to GCA remains unclear. Prior studies have underscored the role of VAP‐1 in granulomatosis with polyangiitis, promoting immune cell adhesion and endothelial dysfunction [7]. Moreover, VAP‐1 has been shown to drive proinflammatory IL‐6 signaling and angiogenesis in endothelial models [8]. Beyond its membrane‐bound form, sVAP‐1 has been implicated in chronic inflammatory diseases due to its enzymatic and signaling functions [5]. However, despite evidence linking sVAP‐1 to chronic inflammatory conditions [5], its concentrations were not significantly elevated in our study, though sVAP‐1 was positively associated with CRP contrasting prior data [9]. Interestingly, MMP‐9 was significantly increased in GCA patients, aligning with its role in both VAP‐1 cleaving and GCA pathophysiology [4]. Flow cytometry data further revealed a significant upregulation of Siglec‐9 across multiple immune cell subsets, indicating a broader immunological role beyond its previously established association with neutrophils and monocytes [10].

Several limitations should be acknowledged. Although the flow cytometry findings add a valuable immunological perspective, they remain exploratory, and no final mechanistic conclusions can be drawn. Moreover, the study was not designed to directly compare [⁶⁸Ga]Ga‐DOTA‐Siglec‐9‐PET/CT with established tracers such as [¹⁸F]FDG‐PET/CT. Future studies should evaluate [⁶⁸Ga]Ga‐DOTA‐Siglec‐9 uptake in age‐matched healthy controls, patients with atherosclerosis, and GCA patients in clinical remission to determine whether these markers can reliably distinguish active vasculitis from chronic vascular remodeling.

To conclude, our results suggest that [^68^Ga]Ga‐DOTA‐Siglec‐9‐PET/CT not only detects vascular inflammation in GCA but also may overcome the limitations of established diagnostics in assessing disease activity. Additionally, we raise questions about the potential pathophysiological roles as biomarkers of VAP‐1 and Siglec‐9 in GCA. Further research with a larger sample size is warranted, with the potential to influence current diagnostic approaches.

## Author contributions

**Simon M. Petzinna**: Conceptualization; methodology; data curation; investigation; validation; formal analysis; supervision; visualization; project administration; writing—original draft; writing—review and editing. **Jim Küppers**: Conceptualization; methodology; validation; investigation; supervision; project administration; writing—original draft; writing—review and editing; data curation; formal analysis; visualization. **Benedikt Schemmer**: Methodology; data curation; investigation; validation; formal analysis; visualization; writing—review and editing; conceptualization. **Anna L. Kernder**: Investigation; data curation; validation; formal analysis; conceptualization; methodology; writing—review and editing; visualization. **Claus‐Jürgen Bauer**: Validation; investigation; writing—review and editing. **Niklas T. Baerlecken**: Validation; investigation; data curation; writing—review and editing; formal analysis. **Denada Bruci**: Validation; investigation; formal analysis; writing—review and editing. **Pantelis Karakostas**: Investigation; writing—review and editing. **Raúl N. Jamin**: Validation; formal analysis; investigation; resources; data curation; writing—review and editing; visualization. **Maike S. Adamson**: Validation; formal analysis; investigation; writing—review and editing. **Anja Winklbauer**: Validation; investigation; data curation; writing—review and editing. **Rayk Behrendt**: Investigation; validation; writing—review and editing. **Markus Essler**: Conceptualization; methodology; data curation; investigation; validation; formal analysis; supervision; visualization; project administration; resources; writing—original draft; writing—review and editing. **Valentin S. Schäfer**: Conceptualization; methodology; data curation; investigation; validation; formal analysis; supervision; visualization; project administration; resources; writing—original draft; writing—review and editing.

## Conflict of interest statement

The authors declare no conflicts of interest.

## Funding information

This research received no external funding.

## Ethics statement

The case series was conducted in accordance with the Declaration of Helsinki and received approval from the ethics committee of the University Hospital Bonn, Germany (reference number: 321/22). Written informed consent was obtained from the patient prior to inclusion.

## Patient involvement and informed consent statement

This project was discussed and reviewed in collaboration with patient representatives as part of the Patient Advisory Board of the Department of Rheumatology at the University Hospital of Bonn. Informed consent was obtained from all patients involved in the study.

## Supporting information




**Table S1**. Demographic, clinical, laboratory, and imaging data in giant cell arteritis patients with relapse and healthy controls.
**Table S2**. Relative tracer uptake in [^68^Ga]Ga‐DOTA‐Siglec‐9‐PET/CT in giant cell arteritis patients with relapse.
**Table S3**. Enzyme‐linked immunosorbent assay data in giant cell arteritis patients with relapse and healthy controls.
**Table S4**. Relative expression of Siglec‐9 on various cell lines in giant cell arteritis patients with relapse.
**Figure S1**. Pathophysiological role of VAP‐1 and its radioactive labeling by the [^68^Ga]Ga‐DOTA‐Siglec‐9 tracer.
**Figure S2**. Enzyme‐linked immunosorbent assay results in relapsing giant cell arteritis patients and healthy controls.
**Figure S3**. Flow cytometry analysis of relative Siglec‐9 expression in various immune cell subsets in giant cell arteritis relapse patients and healthy controls.

## Data Availability

Data are available upon reasonable request from the corresponding author.
