# Supplementary material for: Advanced imaging of relapse in giant cell arteritis: The role of vascular adhesion protein‐1 and [68Ga]Ga‐DOTA‐Siglec‐9 positron emission tomography–computed tomography
Source: J Intern Med. 2025 Jun 26;298(2):138–42. doi: 10.1111/joim.20111 (PMC12239052; doi:10.1111/joim.20111)
Supplement: Supplementary file 1 — Table S1. Demographic, clinical, laboratory, and imaging data in giant cell arteritis patients with relapse and healthy controls. Table S2. Relative tracer uptake in [68Ga]Ga‐DOTA‐Siglec‐9‐PET/CT in giant cell arteritis patients with relapse. Table S3. Enzyme‐linked immunosorbent assay data in giant cell arteritis patients with relapse and healthy controls. Table S4. Relative expression of Siglec‐9 on various cell lines in giant cell arteritis patients with relapse. Figure S1. Pathophysiological role of VAP‐1 and its radioactive labeling by the [68Ga]Ga‐DOTA‐Siglec‐9 tracer. Figure S2. Enzyme‐linked immunosorbent assay results in relapsing giant cell arteritis patients and healthy controls. Figure S3. Flow cytometry analysis of relative Siglec‐9 expression in various immune cell subsets in giant cell arteritis relapse patients and healthy controls. [file JOIM-298-138-s001.docx]

**Supplementary Tables**:

|  | **Age at diagnosis (y)** | **Sex** | **Time since diagnosis (m)** | **LVV involve-ment at diagnosis** | **Cranial involve-ment at diagnosis** | **PMR at diag-nosis** | **CRP at diag-nosis (m/l)** | **OGUS at diagnosis** | **OGUS prior to relapse** | **OGUS at relapse** | **PPE (mg/ 7 days)** | **CRP at relapse/ inclusion (mg/l)** |
| --- | --- | --- | --- | --- | --- | --- | --- | --- | --- | --- | --- | --- |
| **A** | 75.1 | m | 13.1 | no | yes | no | 65.8 | 1.51 | 0,98 | 0,90 | 140 | 8.9 |
| **B** | 68.6 | w | 14.4 | yes | no | no | 26.2 | 0.61 | 0,70 | 1,03 | 0 | 33.6 |
| **C** | 73.5 | w | 36.1 | no | yes | yes | 53.7 | 0.96 | 0,99 | 0,68 | 70 | 112.3 |
| **D** | 85.9 | m | 66.5 | yes | yes | no | 54.2 | 0.80 | 0,60 | 0,94 | 42 | 21.3 |
| **E** | 54.6 | w | 25.1 | yes | no | no | 20.0 | 0.58 | 0,59 | 0,94 | 0 | 16.1 |
| **F** | 66.7 | w | 16.8 | yes | no | yes | 34.8 | 0.95 | 1,04 | 0,74 | 0 | 15 |
| **G** | 67.8 | w | 8.6 | yes | yes | yes | 151.9 | 1.45 | 0,73 | 1,19 | 7 | 114.6 |
| **H** | 82.3 | m | 21.9 | yes | yes | yes | 38.0 | 1.22 | 0,98 | 0,90 | 35 | 32.2 |
| **HC** | 73.6 | w | N/A | N/A | N/A | N/A | N/A | N/A | N/A | N/A | 0 | 0.6 |
| **HC** | 67.1 | w | N/A | N/A | N/A | N/A | N/A | N/A | N/A | N/A | 0 | 0.7 |
| **HC** | 53.1 | w | N/A | N/A | N/A | N/A | N/A | N/A | N/A | N/A | 0 | 0.6 |
| **HC** | 66.0 | m | N/A | N/A | N/A | N/A | N/A | N/A | N/A | N/A | 0 | 6.2 |
| **HC** | 67.1 | w | N/A | N/A | N/A | N/A | N/A | N/A | N/A | N/A | 0 | 0.6 |
| **HC** | 90.6 | m | N/A | N/A | N/A | N/A | N/A | N/A | N/A | N/A | 0 | 0.6 |
| **HC** | 52.4 | m | N/A | N/A | N/A | N/A | N/A | N/A | N/A | N/A | 0 | 1.5 |
| **HC** | 58.7 | w | N/A | N/A | N/A | N/A | N/A | N/A | N/A | N/A | 0 | 1.6 |

***sTable 1: Demogarphic, Clinical, Laboratory and Imaging Data in Giant Cell Arteritis Patients with Relapse and Healthy Controls***

*This table presents the demographic, clinical, laboratory and imaging data of eight patients experiencing relapses of giant cell arteritis and eight healthy controls. CRP levels are shown to be significantly elevated in relapse patients compared to healthy controls. The labels A-H correspond to the patient numbers listed in Figure 1. Institutional upper limit of normal for CRP was 5 mg/L. Abbrv.: CRP: C-reactive protein, LVV: large vessel vasculitis, OGUS: Outcome Measures in Rheumatology (OMERACT) GCA ultrasonography score (OGUS), PMR: polymyalgia rheumatica PPE: prior prednisolone exposure, N/A: not available, HC: healthy control.*

|  | **Aortic arch** | **Subclavian artery (r)** | **Axillary artery (r)** | **Brachialis artery (r)** | **Subclavian artery (l)** | **Axillary artery (l)** | **Brachialis artery (l)** | **Descendening aorta.** | **Thoracic aorta.** | **Abdominal aorta** |
| --- | --- | --- | --- | --- | --- | --- | --- | --- | --- | --- |
| **A** | 2.61 | 2.41 | 2.54 | 1.79 | 2.44 | 2.26 | 1.77 | 3.83 | 3.84 | 3.72 |
| **B** | 2.70 | 2.19 | 1.95 | 1.22 | 2.46 | 2.42 | 1.23 | 3.61 | 3.52 | 3.97 |
| **C** | 2.19 | 2.04 | 1.94 | 1.10 | 2.30 | 1.80 | 1.22 | 2.78 | 3.44 | 2.11 |
| **D** | 2.50 | 2.68 | 2.04 | 1.72 | 2.25 | 2.25 | 1.74 | 3.59 | 3.92 | 2.44 |
| **E** | 3.15 | 2.34 | 1.77 | 1.66 | 2.61 | 2.14 | 1.70 | 3.31 | 4.59 | 3.01 |
| **F** | 3.1 | 3.20 | 2.46 | 1.45 | 3.18 | 2.26 | 1.50 | 4.60 | 4.30 | 3.41 |
| **G** | 2.79 | 2.48 | 2.30 | 1.56 | 2.24 | 1.95 | 1.63 | 2.59 | 4.01 | 3.61 |
| **H** | 3.51 | 3.55 | 2.49 | 1.89 | 3.11 | 2.46 | 1.85 | 5.67 | 5.98 | 4.83 |

***sTable 2: Relative Tracer Uptake in [^68^Ga]Ga-DOTA-Siglec-9-PET/CT in Giant Cell Arteritis Patients with Relapse***

*This table shows the relative maximum tracer uptake in different vascular regions of patients with giant cell arteritis during a relapse, as observed in [^68^Ga]Ga-DOTA-Siglec-9-PET/CT scans. The regions measured include the aortic arch, subclavian artery (right and left), axillary artery (right and left), brachialis artery (right and left), descending aorta, thoracic aorta, and abdominal aorta. The labels A-H correspond to the patient numbers listed in Figure 1. Abbrv.: r: right, l: left.*

|  | **sVAP-1 (pg/ml)** | **MMP2 (µg/ml)** | **MMP3 (ng/ml)** | **MMP9 (µg/ml)** |
| --- | --- | --- | --- | --- |
| **A** | 611.4 | 53.6 | 117.9 | 36.9 |
| **B** | 964 | 47.6 | 24.6 | 12.1 |
| **C** | 880.8 | 55.7 | 16.2 | 10.3 |
| **D** | 543.3 | 50.4 | 12.9 | 3.4 |
| **E** | 985.6 | 91.7 | 41.4 | 15.3 |
| **F** | 682.5 | 56.5 | 12.4 | 16.1 |
| **G** | 1147.8 | 58.5 | 35.4 | 25.6 |
| **H** | 743.6 | 63.1 | 42.7 | 43.6 |
| **HC** | 722.8 | 45.31 | 14.5 | 1.8 |
| **HC** | 777.9 | 65.3 | 17.1 | 8.6 |
| **HC** | 565.2 | 69.6 | 15.6 | 3.2 |
| **HC** | 590.5 | 85.4 | 23.5 | 7.0 |
| **HC** | 955.2 | 54.3 | 10.2 | 12.2 |
| **HC** | 980.9 | 188.8 | 17.2 | 5.8 |
| **HC** | 684.5 | 26.8 | 18.7 | 3.6 |
| **HC** | 531.5 | 54.3 | 34.4 | 2.6 |
| **p-value** | .3414 | .4519 | .1498 | **.0110*** |

***sTable 3: Enzyme-linked immunosorbent assay Data in Giant Cell Arteritis Patients with Relapse and Healthy Controls***

*This table presents the enzyme-linked immunosorbent assay data of eight patients experiencing relapses of giant cell arteritis and eight healthy controls. It includes sVAP-1, MMP2, MMP3, and MMP9 in the serum of patients with giant cell arteritis during relapse. MMP9 levels are shown to be significantly elevated in relapse patients compared to healthy controls. *p < 0.05 was considered significant. The labels A-H correspond to the patient numbers listed in Figure 1. Abbrv.: (s)VAP-1: (soluble) vascular adhesion protein-1, MMP: matrix metalloproteinase, HC: healthy control.*

|  | **CD3+/ CD4+** | **CD3+/ CD8+** | **CD14+/ CD16-** | **CD14+/ CD16+** | **CD14-/ CD16+** | **CD19+/ CD38-** | **CD19+/ CD38+** | **CD19-/ CD38+** | **CD16+/ CD38+** |
| --- | --- | --- | --- | --- | --- | --- | --- | --- | --- |
| **A** | 53.8 | 19.1 | 81.2 | 66.8 | 57.6 | 16.7 | 65.9 | 66.9 | 79.0 |
| **B** | 34.5 | 22.6 | 90.7 | 78.1 | 63.1 | 21.7 | 73.3 | 68.0 | 83.2 |
| **C** | 54.2 | 12.0 | 77.9 | 81.4 | 44.3 | 16.1 | 68.8 | 61.3 | 78.4 |
| **D** | 49.0 | 11.6 | 84.4 | 76.2 | 51.2 | 18.0 | 76.8 | 60.6 | 79.4 |
| **E** | 43.8 | 8.39 | 75.9 | 82.2 | 52.9 | 16.6 | 69.9 | 60.5 | 86.0 |
| **F** | 46.8 | 19.8 | 83.3 | 84.6 | 52.1 | 25.6 | 79.3 | 72.9 | 85.0 |
| **G** | 86.7 | 19.5 | 79.4 | 94.6 | 62.1 | 43.0 | 93.3 | 57.5 | 93.8 |
| **H** | 44.0 | 19.7 | 76.8 | 53.2 | 48.9 | 1.67 | 35.2 | 36.0 | 60.9 |
| **HC** | 47.2 | 25.2 | 65.4 | 48.0 | 53.4 | 5.0 | 48.3 | 43.4 | 70.4 |
| **HC** | 45.8 | 12.7 | 88.6 | 49.9 | 49.4 | 0.5 | 16.8 | 9.2 | 56.1 |
| **HC** | 23.4 | 10.2 | 84.8 | 35.3 | 27.6 | 1.4 | 21.7 | 40.3 | 41.2 |
| **HC** | 49.0 | 11.3 | 86.5 | 35.3 | 50.4 | 0.3 | 8.64 | 9.23 | 57.9 |
| **HC** | 48.2 | 16.0 | 85.5 | 61.8 | 41.9 | 1.3 | 15.7 | 23.3 | 54.6 |
| **HC** | 48.1 | 18.5 | 75.0 | 75.0 | 68.1 | 6.0 | 78.3 | 28.6 | 91.9 |
| **HC** | 62.8 | 20.2 | 80.1 | 79.3 | 73.8 | 0.3 | 18.9 | 29.0 | 84.5 |
| **HC** | 47.9 | 23.1 | 85.7 | 56.6 | 53.6 | 0.9 | 32.5 | 47.8 | 59.3 |
| **p-value** | 0.462 | 0.836 | 0.940 | **0.009** | 0.758 | **<0.001** | **<0.001** | **<0.001** | **0.032** |

***sTable 4: Relative Expression of Siglec-9 on Various Cell Lines in Giant Cell Arteritis Patients with Relapse***

*The table shows the frequency of Siglec 9+ cells as percent of the indicated peripheral blood mononuclear cell subsets, including CD3+CD4+ T cells, CD3+CD8+ T cells, CD14+CD16- classical monocytes, CD14+CD16+ intermediate monocytes, CD14-CD16+ non-classical monocytes, CD19+CD38+ plasmablast, CD19-CD38+ plasmacells, CD19+CD38- naïve B cells, and CD16+CD38+ natural killer cells. The labels A-H correspond to the patient numbers listed in Figure 2. *p < 0.05 was considered significant. Abbrv.: CD: cluster of differentiation, HC: healthy control.*

**Supplementary Figures**

***
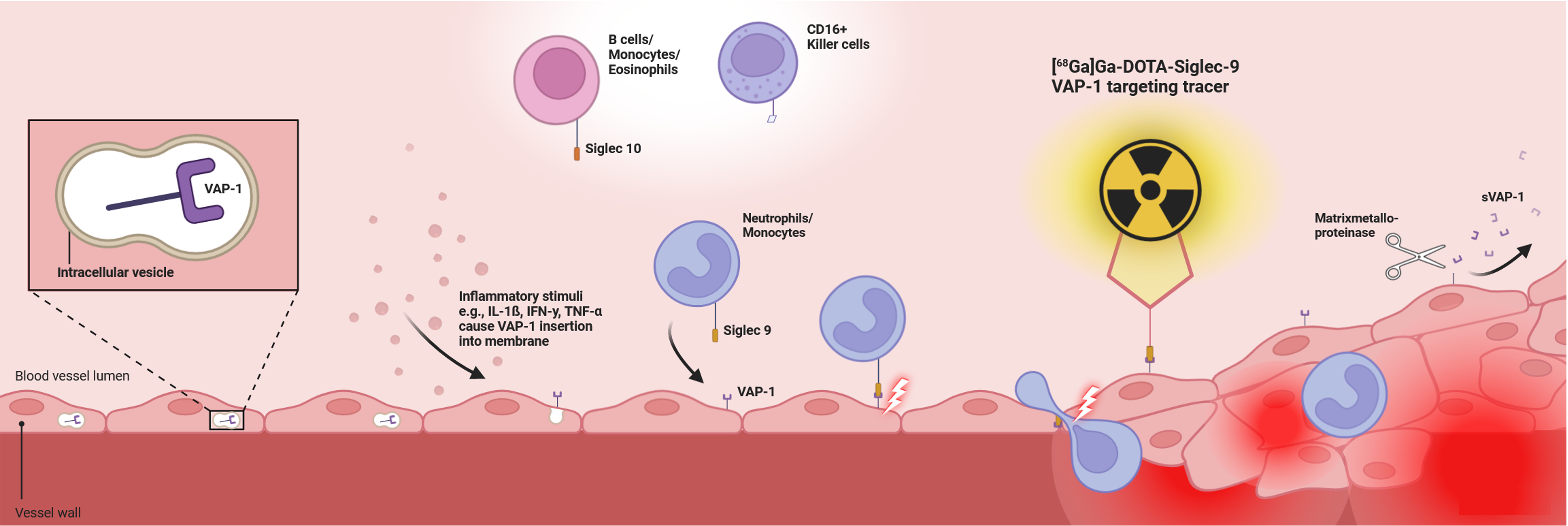
 sFigure 1: Pathophysiological Role of VAP-1 and its Radioactive Labeling by the [^68^Ga]Ga-DOTA-Siglec-9 Tracer***

*The first part of the figure schematically illustrates the endothelial translocation of VAP-1 from intracellular vesicles following inflammatory stimuli, along with the subsequent binding of neutrophils and monocytes via the Siglec-9 ligand, while the second part depicts the [^68^Ga]Ga-DOTA-Siglec-9 radiotracer bound to endothelially expressed VAP-1. Created with BioRender.com.* *Abbrv.: (s)VAP-1: (soluble) vascular adhesion protein 1, Siglec 9: sialic acid-binding immunoglobulin-like lectin-9, CD: cluster of differentiation, IL: interleukin, IFN: interferon, TNF: tumor necrosis factor.*

*
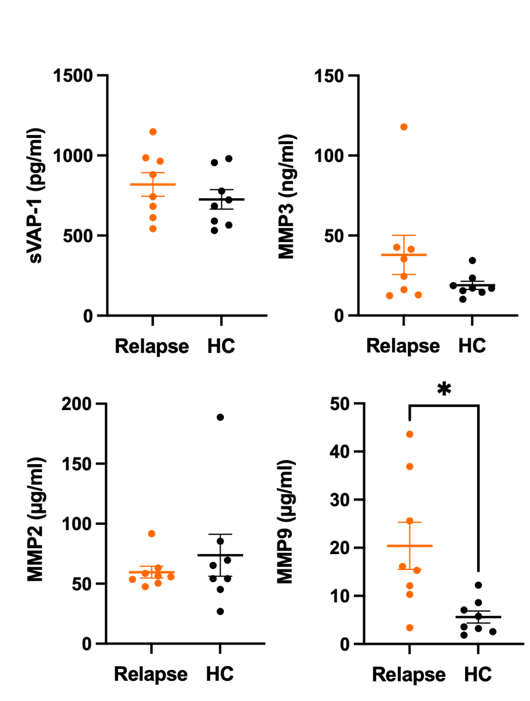
*

***sFigure 2: Enzyme-Linked Immunosorbent Assay Results in Relapsing Giant Cell Arteritis Patients and Healthy Controls***

*This figure illustrates the results of laboratory analysis conducted to evaluate the levels of sVAP-1, CRP, MMP2, MMP3, and MMP9 in the serum of patients with giant cell arteritis during relapse, compared to healthy controls. MMP9 and CRP levels are shown to be significantly elevated in relapse patients compared to healthy controls. *p < 0.05 was considered significant. Abbrv.: (s)VAP-1: (soluble) vascular adhesion protein-1, CRP: C-reactive protein, MMP: matrix metalloproteinase, HC: healthy control.*

*
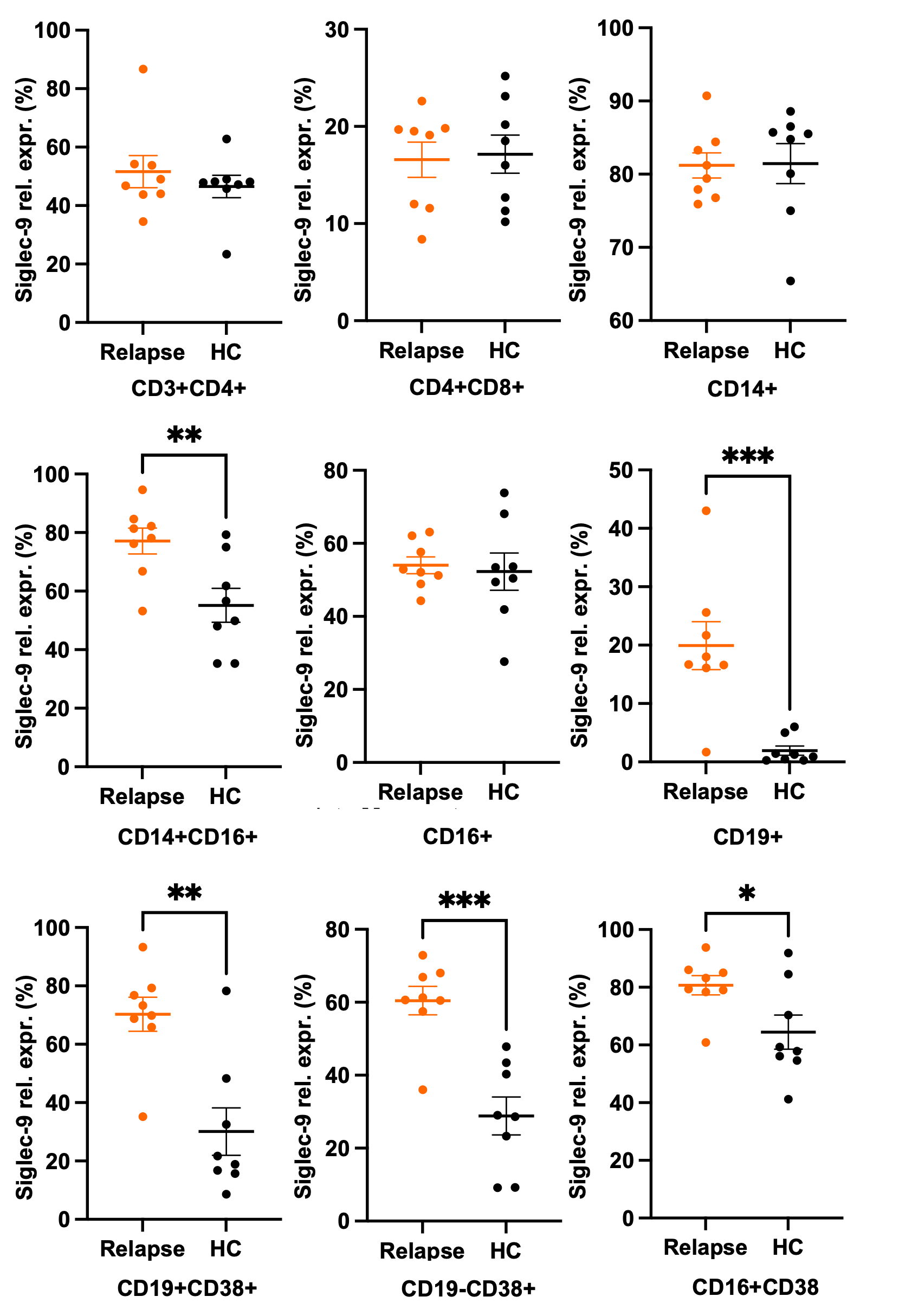
*

***sFigure 3: Flow Cytometry Analysis of Relative Siglec-9 Expression in Various Immune Cell Subsets in Giant Cell Arteritis Relapse Patients and Healthy Controls***

*This figure illustrates the results of flow cytometry analysis conducted to evaluate Siglec-9 expression on immune cell subsets in patients with giant cell arteritis during relapse. The analyzed cell subsets include CD3+CD4+ T cells, CD3+CD8+ T cells, CD14+CD16- classical monocytes, CD14+CD16+ intermediate monocytes, CD14-CD16+ non-classical monocytes, CD19+CD38+ plasmablasts, CD19-CD38+ plasmacells, CD19+CD38- naïve B cells, and CD16+CD38+ natural killer cells. Flow cytometry analysis revealed significantly elevated relative Siglec-9 expression across various immune cell subsets in GCA relapse patients compared to healthy controls. *p < 0.05 was considered significant. Abbrv.: (s)VAP-1: (soluble) vascular adhesion protein-1, CRP: C-reactive protein, CD: cluster of differentiation.*

**Supplementary Protocol***:*

*[^68^Ga]Ga-DOTA-Siglec-9-PET/CT – Radiosynthesis and Imaging*

Gallium-68 was obtained from a germanium-68/gallium-68 generator (EZAG, Berlin, Germany). Synthesis was performed utilizing an automated cassette module (GAIA; Elysia‑Raytest, Straubenhardt, Germany). Standard fluidic and reagent kit for gallium-68 radiolabeling of peptides (ABX advanced biochemical compounds GmbH, Radeberg, Germany) were employed. TraceSelect water and ethanol Ph. Eur. were purchased from Merck (Darmstadt, Germany).

DOTA-Siglec-9 was obtained from ABX and diluted with TraceSelect water to achieve a final concentration of 1 mg/mL. For the synthesis, the module was equipped with 150 ± 5.00 µg DOTA-Siglec-9, 500 µL eluent, 2.49 mL ammonium acetate buffer (pH 4.5) and 200 µL ethanol. After radiolabeling at 65 ± 5 °C for 15.00 min, the reaction mixture was purified through C-18 solid phase extraction and the final product was obtained in isotonic sodium chloride solution.

For quality control, an aliquot of about 50 µl was taken from the final formulation before measurement of radioactivity. All chemicals were pure or analytical grade and used as received, unless otherwise specified.

Radioactivity of the final product was measured with a dose calibrator (ISOMED 2010, MED Nuklear-Medizintechnik Dresden GmbH, Dresden, Germany). Radiochemical purity was determined using glass microfiber chromatography paper impregnated with silica-gel (iTLC‑SG, Agilent Technologies, Santa Clara, USA) and analyzed with a single trace radioTLC‑scanner (PET-miniGita, Elysia-Raytest, Straubenhardt, Germany) and evaluation software (Gina Star TLC, Elysia-Raytest, Straubenhardt, Germany). iTLC-strips were developed in 0.1 M citric buffer (pH 4; Merck, Darmstadt, Germany) and 1 M ammonium acetate/methanol (1:1) (v:v). Furthermore, radioHPLC was used to determine the radiochemical purity and identification of the product species. RadioHPLC was performed utilizing Agilent 1260 Infinity II reverse phase HPLC system (Agilent Technologies, Santa Clara, USA) equipped with Gabi γ-HPLC flow detector (Elysia-Raytest, Straubenhardt, Germany) and a PC interface running Gina Star (Elysia-Raytest, Straubenhardt, Germany). A Nucleodur 100-3 C18 ec 125/4 column (Macherey-Nagel GmbH & Co. KG, Düren, Germany) was applied. The gradient utilized mobile phase A (deionized water + 0.1% TFA) and mobile phase B (acetonitrile + 0.1% TFA) at a flow rate of 0.7 mL/min starting with 100% A / 0% B to 0% A / 100% B within 20 min. pH was measured using pH-indicator strips MColorpHast 2.0−9.0 (Merck, Darmstadt, Germany). The approximate half-life of gallium-68 was determined using a dose calibrator (ISOMED 2010, MED Nuklear-Medizintechnik Dresden GmbH, Dresden, Germany). The nuclide specific energy of gallium-68 as well as germanium-68 breakthrough (48h post labeling) were measured using a multi‑channel‑analyzer for γ‑spectroscopy (MUCHA; Elysia-Raytest, Straubenhardt, Germany). Clear appearance was checked visually. Filter integrity was tested with GAIA (Elysia-Raytest, Straubenhardt, Germany). Non-decay corrected (AY) as well as decay corrected radiochemical yield were calculated based on the activity trapped on the SCX, activity trapped on C-18 and remaining activity on C-18 after final formulation as measured by the module. Volume activity and apparent molar activity were calculated based on activity of the final product.

Radiosynthesis of [^68^Ga]Ga-DOTA-Siglec-9 with 1.55 ± 0.17 GBq gallium-68 revealed a decay corrected radiochemical yield of 90.0 ± 4.75% (73.8 ± 3.92% AY). A volume activity of 126 ± 21.0 MBq/ml and an apparent molar activity of 20.3 ± 3.32 MBq/nmol were obtained. Radiochemical purity was > 98%.

The patients underwent [^68^Ga]Ga-DOTA-Siglec-9 PET-CT scans with an injected dose of 135.1 ± 31.7 MBq [^68^Ga]Ga-DOTA-Siglec-9. A low-dose CT for attenuation correction and a consecutive whole-body PET scan were performed 56.2 ± 8.3 minutes post-injection. A Vereos PET-CT scanner (Philips, Amsterdam, Netherlands) was utilized, which is equipped with digital photon counting detectors. The transaxial and axial fields of view were 67.6 and 16.4 cm, respectively. Emission data was acquired for 60 s per bed. Images were reconstructed using the OSEM algorithm. Both the time-of-flight algorithm and point spread function correction were applied. The reconstructed images had a matrix size of 256 × 256 mm and a voxel size of 2.0 × 2.0 × 2.0 mm. To compensate for noise and random effects a single thick coronal MIP (maximum intensity projection) with a thickness of 240 mm was created for each patient. Activity in the vessels was measured in terms of the SUVmax in a circular ROI (region of interest). This represents the value of the most intense pixel in that representative ROI along a specific section of a vessel which was chosen in correlation with the low dose CT. SUVmax was calculated as SUVmax = maximum tracer uptake in ROI / (injected activity / patient weight) analog to the newest QIBA profile (Version 1.14) for [^18^F]FDG-PET/CT. This allowed for the exclusion of low uptake from areas of adjacent uninvolved structures. However, the region of interest had to be manually selected with care to include only structures of interest, which was especially important when structures like the bladder or kidneys with high unspecific uptake were adjacent to the vessels.
